# Global phylogeography and genomic characterization of *bla*_NDM-1_-positive clinical *Proteus mirabilis* isolates from China

**DOI:** 10.1128/msystems.00758-25

**Published:** 2025-09-22

**Authors:** Jingyi Guo, Haotian Xu, Chengjin Wu, Hongyan Yu, Lexuan Yang, Yan Qi, Xiuyun Zuo, Hongyin Yang, Linyue Zhang, Yunsong Yu, Xi Li

**Affiliations:** 1 Laboratory Medicine Center, Department of Clinical Laboratory, Zhejiang Provincial People’s Hospital, Affiliated People’s Hospital, Hangzhou Medical College 117839, Hangzhou, China; 2 School of Medical Technology and Information Engineering, Zhejiang Chinese Medical University 70571, Hangzhou, China; 3 The Key Laboratory of Interventional Pulmonology of Zhejiang Province, Department of Pulmonary and Critical Care Medicine, The First Affiliated Hospital of Wenzhou Medical University 89657, Wenzhou, Zhejiang, China; 4 Medical Laboratory Center, Hangzhou TCM Hospital Affiliated to the Zhejiang Chinese Medical University 194033 , Hangzhou, Zhejiang, China; 5 Department of Clinical Laboratory, Feicheng Hospital of Traditional Chinese Medicine, Feicheng, Shandong, China; 6 Department of Clinical Laboratory, Sir Run Run Shaw Hospital, Zhejiang University 12377, Hangzhou, China; 7 Key Laboratory of Precision Medicine in Diagnosis and Monitoring Research of Zhejiang Province, Hangzhou, China; 8 Zhejiang Provincial Engineering Research Center for Innovative Pathogen Precision Detection Instruments, Hangzhou, China; Katholieke Universiteit Leuven, Leuven, Belgium

**Keywords:** carbapenem-resistant *Proteus mirabilis*, *bla*_NDM-1_, genomic epidemiology, Salmonella genomic island 1

## Abstract

Carbapenem-resistant *Proteus mirabilis* (CRPM), largely driven by dissemination of *bla*_NDM-1_, poses a growing global threat, yet its phylogeographic and genetic patterns remain understudied. We analyzed 16 *bla*_NDM-1_-harboring CRPM clinical isolates from a tertiary hospital in China (2017–2024) using antimicrobial susceptibility testing, conjugation assays, whole-genome sequencing, and growth experiments. We found that *bla*_NDM-1_ dissemination occurred primarily via plasmids and Salmonella Genomic Island 1 (SGI1), including a novel SGI1-PM16 variant. Early transmission events were associated with Tn*125*-derived elements (ΔTn*125*), while IS*CR1* appeared to mediate rolling-circle transposition and likely facilitated local amplification of resistance cassettes. Additionally, we performed a global genomic epidemiological study of 420 *bla*_NDM_-positive CRPM genomes curated from NCBI (accessed 21 April 2025). ST135 emerged as the predominant clone among CRPM in China. Phylogeographic analysis of *P. mirabilis* worldwide clarified the geographic prevalence of NDM variants. In the United States, *bla*_NDM-7_ predominates, whereas *bla*_NDM-1_ is most frequent in China. In summary, this study provides crucial insights into the resistance mechanisms and transmission dynamics of CRPM, as well as underscores the need to enhance genomic surveillance and optimize infection control strategies to mitigate the spread of *bla*_NDM-1_-harboring CRPMs.

**IMPORTANCE:**

To date, the phylogeographic distribution of *bla*_NDM_-carrying CRPMs has not been determined. Our study identified ST135 as the predominant *bla*_NDM_-producing clone, with a distinct global distribution pattern of NDM variants. Furthermore, we elucidated critical *bla*_NDM-1_ transmission mechanisms, revealing both plasmid- and SGI1-mediated dissemination and IS*CR1*-driven gene amplification, while also characterizing a novel SGI1-PM16 variant. These findings provide significant new insights into the molecular mechanisms underlying acquired antimicrobial resistance.

## INTRODUCTION

With the prevalence of multidrug-resistant (MDR) bacteria, carbapenems are often the last-line agents for severe Gram-negative infections (1, 2). Prolonged or inappropriate antibiotic use has favored emergence of carbapenemase-producing strains that hydrolyze carbapenems (3). Based on an analysis of the global spread of Carbapenem-resistant *Enterobacterales* (CRE), globally, KPC and NDM are the predominant carbapenemases (4, 5). A multicenter study demonstrated that the 30-day mortality rate among patients infected with *bla*_NDM_-carrying CRE was strikingly elevated, reaching 29.7% (6). This poses a significant challenge in the clinical management of Gram-negative bacterial infections.

NDM is a class B metallo-β-lactamase that confers resistance to most β-lactams except monobactams (7). The global spread of the *bla*_NDM_ gene is a major public health concern; *bla*_NDM_ can be transmitted horizontally in different species flanked by transferable DNA vectors, such as conjugative plasmids, integrative conjugative elements, and mobile genetic elements (MGEs) (8–10). The spread of resistance genes is largely attributed to horizontal transfer of plasmids. However, some genetic constructs can be transmitted via mobilization. This is the case for Salmonella genomic island 1 (SGI1), which comprises a conserved backbone and a multidrug-resistance region, where various ARGs are clustered in a complex class 1 integron (11). Recently, SGI1 and its derivatives have been widely reported in *P. mirabilis* (10, 12, 13).

*Proteus mirabilis* is a Gram-negative enterobacterium that frequently causes healthcare-associated urinary tract infections, particularly with prolonged catheter use (14). Carbapenemase emergence is concerning because *P. mirabilis* is intrinsically resistant to polymyxins and tigecycline (15). In this study, we performed a comprehensive molecular and genomic epidemiological analysis of *bla*_NDM-1_-positive carbapenem-resistant *Proteus mirabilis* (CRPM) clinical isolates collected from a tertiary hospital in China between 2017 and 2024. Furthermore, we investigated the phylogenetic relationships, geographic distribution, and population dynamics of NDM-positive CRPMs on a global scale. Our findings provide an in-depth genomic characterization of Chinese CRPM isolates and elucidate their epidemiological connections within the broader global context.

## RESULTS

### Characteristics of the CRPM strains

A total of 170 multidrug-resistant (MDR) *P. mirabilis* clinical isolates were collected from a tertiary hospital in Hangzhou between January 2017 and September 2024. Molecular screening identified 55 *bla*_KPC_-producing and sixteen *bla*_NDM-1_-positive CRPM isolates from this collection. The remaining strains tested negative for all known carbapenem resistance-associated genes. The present study primarily focuses on the genomic characterization of the 16 *bla*_NDM-1_-producing CRPM isolates. These isolates were collected from patients ranging in age from 14 to 96 years (Table 1). Most isolates were obtained from feces (31.3%, 5/16), sputum (25.0%, 4/16), and urine (25.0%, 4/16), while the remaining isolates were derived from blood, ascites, and pharyngeal swabs (6.3% each, 1/16) (Table 1). All isolates were from unique patients except PM33 and PM54, which were from the same individual. PM33 and PM54, although derived from the same specimen, were isolated from specimens collected more than two months apart. All isolates harbored *bla*_NDM-1_.

**TABLE 1:** Biosample information of 16 *bla*_NDM-1_-positive *P. mirabilis* strains recovered from 15 patients

Strain	Patient	Age	Year	Sex	Specimen	Diagnosis
PM14	1	14	2017	Male	Feces	Pancytopenia
PM16	2	79	2017	Male	Ascites	Rectal tumor
PM22	3	88	2019	Female	Urine	Hypertensive disease
PM23	4	55	2020	Male	Sputum	Cervical spinal cord injury
PM32	5	92	2021	Male	Blood	Elevated tumor markers
PM33	6	70	2022	Male	Sputum	Coma
PM34	7	89	2022	Female	Urine	Pulmonary space-occupying disease
PM54	6	70	2022	Male	Sputum	Coma
PM65	8	85	2019	Female	Urine	Hypertensive disease
PM1700	9	91	2023	Male	Feces	Cerebrovascular hemiplegia
PM8086	10	76	2023	Male	Feces	Septic shock
PM7752	11	89	2023	Female	Urine	Cavernous cerebral infarction
PM8747	12	61	2024	Male	Sputum	Hemiplegia
PM0259	13	96	2024	Male	Feces	Severe pneumonia
PM0460	14	50	2024	Male	Throat swab	Hemiplegia
PM3393	15	56	2024	Female	Feces	Coma

AST showed that all *bla*_NDM-1_-positive CRPMs were resistant to meropenem, imipenem, ertapenem, ceftazidime, ceftazidime-avibactam, cefepime, and ciprofloxacin and susceptible to cefiderocol and aztreonam-avibactam. In contrast, only 37.5% of the strains (*n* = 6) were resistant to amikacin, 8 strains were susceptible to amikacin, and 2 isolates were intermediate (Table 2).

**TABLE 2:** Antimicrobial susceptibilities of 16 *P*. *mirabilis* strains and 8 transconjugants conducted by *P. mirabilis* and *E. coli* J53

Minimum inhibitory concentrations (MICs, μg/mL)										
Strain	MEM	IPM	AMK	FEP	CIP	ETP	CAZ	CZA^*a*^	CFDC	AZA^*a*^
Clinical isolates										
PM14	128	128	64	128	16	128	>128	>128	0.25	<0.032
PM16	32	128	8	128	32	64	>128	>128	0.25	<0.032
PM22	64	>128	4	64	32	128	>128	>128	0.25	<0.032
PM23	128	>128	4	64	32	128	>128	>128	0.25	<0.032
PM32	>128	>128	4	>128	32	>128	>128	>128	2	<0.032
PM33	64	>128	4	>128	32	128	>128	>128	0.25	<0.032
PM34	32	128	4	128	32	64	>128	>128	0.25	<0.032
PM54	64	128	16	>128	32	64	>128	>128	1	<0.032
PM65	64	128	32	128	32	64	>128	>128	2	<0.032
PM1700	128	128	8	128	8	128	>128	>128	1	<0.032
PM8086	64	128	16	128	32	64	>128	>128	0.25	<0.032
PM7752	32	128	>128	64	32	64	>128	>128	0.25	<0.032
PM8747	64	128	32	128	32	64	>128	>128	0.125	<0.032
PM0259	32	128	4	>128	32	16	>128	>128	4	<0.032
PM0460	64	>128	4	128	16	128	>128	>128	1	<0.064
PM3393	64	>128	2	64	16	>128	>128	>128	0.5	<0.032
Transconjugants										
JPM14_NDM	32	32	2	128	<0.125	>128	>128	>128	–	–
JPM22_NDM	64	16	2	128	<0.125	>128	>128	>128	–	–
JPM65_NDM	8	8	32	16	<0.125	64	>128	>128	–	–
JPM8747_NDM	8	4	32	32	<0.125	32	>128	>128	–	–
JPM8086_NDM	32	16	64	64	<0.125	>128	>128	>128	–	–
JPM1700_NDM	128	128	2	128	0.5	>128	>128	>128	–	–
JPM3393_NDM	32	8	1	64	0.5	>128	>128	>128	–	–
JPM0460_NDM	8	4	1	32	<0.125	128	>128	>128	–	–
J53	<0.125	0.25	2	0.25	<0.125	<0.125	0.25	<0.125	–	–
AT25922	<0.125	0.25	2	<0.125	<0.125	<0.125	0.25	<0.125	<0.032	<0.032

^*a*^ Avibactam was added at 4 µg/mL. MEM, Meropenem; IPM, Imipenem; ETP, Ertapenem; AMK, Amikacin; FEP, Cefepime; CIP, Ciprofloxacin; CAZ, Ceftazidime; CZA, Ceftazidime-avibactam; CFDC, Cefiderocol; AZA, Aztreonam-avibactam. The "–" in the table indicates that the item was not detected.

### Genomic characteristics of CRPM strains harboring *bla*_NDM-1_

**Fig 1:**
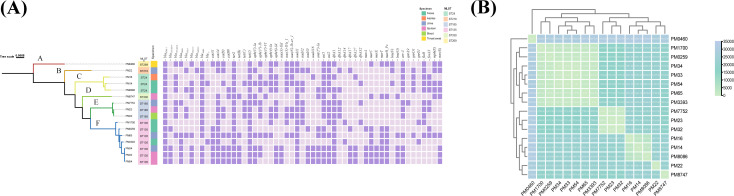
(**A**) Phylogenetic relationship of the *bla*_NDM-1_ producing *P. mirabilis* in this study. The purple squares represent the corresponding resistance genes. (**B**) Heatmap visualization of SNPs in 16 *bla*_NDM-1_-positive *P. mirabilis* clinical isolates.

WGS analysis of all 16 CRPM isolates revealed a conserved chromosomal size (~4 Mb) and a variable plasmid content (0–3 plasmids per isolate). To delineate phylogenetic relationships and genomic epidemiology among NDM-producing CRPMs, we performed high-resolution molecular characterization using multilocus sequence typing (MLST) and core genome phylogenetic analysis. Phylogenetic reconstruction resolved the NDM-1-producing isolates into six distinct clades (Fig. 1A), with corresponding stratification into six sequence types (STs): ST135 (44%, *n* = 7/16), ST204 (19%, *n* = 3/16), ST185 (19%, *n* = 3/16), ST218 (6%, *n* = 1/16), ST330 (6%, *n* = 1/16), and ST269 (6%, *n* = 1/16). SNP identification revealed that the three isolates (PM32, PM33, and PM54) were highly homologous (pairwise core-genome SNP distance <13) (Fig. 1B), indicating the presence of clonal dissemination among the isolates. Core genome phylogeny further demonstrated clonal clustering of seven isolates within ST135, suggesting a closely related genomic lineage. In contrast, the remaining nine isolates exhibited broad genetic diversity, distributed across five divergent STs (ST185, ST204, ST218, ST330, and ST269), indicating polyclonal dissemination of NDM-1 among these strains.

WGS identified 30 distinct antimicrobial resistance genes (ARGs) across the 16 isolates spanning 9 antibiotic classes. These include β-lactams (*bla*_NDM-1_, *bla*_CTX-M-14_, *bla*_CTX-M-65_, *bla*_TEM-1_, *bla*_OXA-10_, and *bla*_OXA-1_), chloramphenicols (*catA1*, *catA4*, *catB3*, *catB8,* and *floR*), aminoglycosides [*aph(3')-Ia*, *aph(3'')-Ib*, *aph (4)-Ia*, *aph (6)-Id*, *aac (3)-IId*, *aac (3)-IVa_1,* and *ant(2'')-Ia*], tetracyclines [*tet(B*), *tet(C*), *tet(J*), and *tet(Z*)], sulfonamides (*sul1* and *sul2*), fosfomycin (*fosA3*), macrolides [*ere(A*) and *mph(E*)], fluoroquinolones [*aac(6')-Ib-cr_1*], lincoamides [*lnu(F*) and *lnu(G*)], and rifamycins (*arr-3*) (Table 3; Fig. 1A). In four isolates, *bla*_NDM-1_ was chromosomal within SGI1. In contrast, the *bla*_NDM-1_ gene of the remaining isolates was located on the IncN (*n* = 2), pPrY2001-like plasmids (*n* = 6), and pPM54-NDM_252k-like plasmids (*n* = 3). Notably, PM8086 carried *bla*_NDM-1_ on both the chromosome (SGI1) and a pPrY2001-like plasmid (Table 3). In addition, *bla*_NDM-1_ was single-copy in most isolates, with the exception of PM32, which has an additional *bla*_NDM-1_ copy on the SGI (Table 3).

**TABLE 3:** The genome characteristics of 16 *P*. *mirabilis* isolates in this study^*a*^

Strain IDs	No. of plasmids	Chromosome or plasmids	GenBank accession	Size (bp)	Plasmid type	Resistance genes
PM14	4	Chromosome	CP137085	4,118,536	/	*terZ, tet(J), aadA2, ere(A), dfrA32, bleO, sul1, arr-3, catB3, bla*_OXA-1_ *, aac(6')-Ib-cr_1, aac(6')-Ib-cr_1, aph (4)-Ia, sul2, floR, bla*_CTX-M-65_ *, fosA3, aph(3')-Ia, tet(C), aph(3'')-Ib, aph (6)-Id, aph(3')-VIa*
		pPM14-2	CP137086	4,223	Col3M	*/*
		pPM14-3	CP137084	2,683	Col3M	*qnrD1*
		pPM14-NDM_123k	CP137087	123,168	/	*lnu(F), aadA1, sul1, ble*_-MBL_*, bla*_NDM-1_*, aph(3')-VI, mph(E), msr(E*)
PM16	3	Chromosome	CP137335	3,971,396	/	*terZ, tet(J), dfrA1, sul2, aph(3'')-Ib, aph (6)-Id, floR, fosA3, bla*_CTX-M-65_ *, aac (3)-IVa_1, aph (4)-Ia, sul1, arr-3, bla*_NDM-1_ *, ble*_-MBL_ *, aadA16, dfrA27, aac(6')-Ib-cr_1*
		pPM16-1	CP137337	8,178	/	*/*
		pPM16-2	CP137336	2,683	Col3M	*qnrD1*
PM22	3	Chromosome	CP137214	3,891,752	/	*catA4, terZ, tet(J), floR, sul2, aph (4)-Ia, aac (3)-IVa_1, aac(6')-Ib-cr_1, bla*_OXA-1_ *, catB3, arr-3, sul1, dfrA12, aadA2, aadA5, dfrA17, bla*_CARB-2_
		pPM22-2	CP137215	2,683	Col3M	*qnrD1*
		pPM22-NDM_109k	CP137216	109,990	pPrY2001-like	*msr(E), mph(E), bla*_NDM-1_*, ble*_-MBL_*, aph(3')-Ia, sul1, aadA1, lnu(F*)
PM23	1	Chromosome	CP137220	4,043,825	/	*catA4, terZ, tet(J), sul1, arr-3, bla*_OXA-1_ *, catB3, aac(6')-Ib-cr_1, floR, aph (6)-Id, aph(3'')-Ib, sul2, tet(C), fosA3, bla*_CTX-M-65_ *, aac (3)-IVa_1, aph (4)-Ia, aadA1, dfrA1, bla*_NDM-1_ *, ble*_-MBL_ *, aadA2, dfrA12*
PM32	1	Chromosome	CP137736	4,042,001	/	*catA4, terZ, tet(J), sul1, arr-3, bla*_NDM-1_ *, bla*_OXA-1_ *, catB3, aac(6')-Ib-cr_1, sul2, aph (4)-Ia, aac (3)-IVa_, bla*_CTX-M-65_ *, tet(C), aph(3'')-Ib, aph (6)-Id, floR, aadA1, dfrA1, bla*_NDM-1_ *, ble*_-MBL_ *, aadA2, dfrA12*
PM33	3	Chromosome	CP137217	4,255,535	/	*bla*_CTX-M-14_ *, catA4, terZ, tet(J), catA1, bla*_TEM-1_ *, aph(3')-Ia, sul2, aph(3'')-Ib, aph (6)-Id, aac (3)-IId, sul1, aadA5, dfrA17, merC, merT, merR_Ps, aadA1, dfrA1*
		pPM33-2	CP137218	52,937	/	*bla*_CTX-M-14_
		pPM33-NDM_252k	CP137219	252,119	pPM54-NDM_252k-like Plasmid	*lnu(G), lnu(G), catB8, bla*_OXA-10_ *, aadA1, dfrA1, sul1, ble*_-MBL_ *, bla*_NDM-1_ *, catB3, arr-3, dfrA27, aadA16, msr(E), merC, merT, merR_Ps, tet(B), merE, merA, merT*
PM34	/	/	JAWQKU000000000	4,467,297	pPM54-NDM_252k-like Plasmid	*aph(3')-Ia, aadA1, dfrA1, catA4, merE, merA, merT, lnu(G), catA1, terZ, tet(J), aadA16, dfrA27, arr-3, catB3, bla*_NDM-1_ *, ble*_-MBL_ *, aac (3)-IId, aph (6)-Id, aph(3'')-Ib, sul2, merC, merT, merR_Ps, bla*_CTX-M-14_ *, tet(B), msr(E), ant(2'')-Ia, catB8, bla*_OXA-10_ *, bla*_TEM-1_ *, dfrA17, aadA5, sul1*
PM54	3	Chromosome	CP137228	4,255,535	/	*bla*_CTX-M-14_ *, catA4, terZ, tet(J), catA1, bla*_TEM-1_ *, aph(3')-Ia, sul2, aph(3'')-Ib, aph (6)-Id, aac (3)-IId, sul1, aadA5, dfrA17, merC, merT, merR_Ps, aadA1, dfrA1*
		pPM54-2	CP137229	52,937	/	*bla*_CTX-M-14_
		pPM54-NDM_252k	CP137230	252,119	pPM54-NDM_252k-like Plasmid	*merT, merA, merE, tet(B), merR_Ps, merT, merC, msr(E), sul1, aadA16, dfrA27, arr-3, catB3, bla*_NDM-1_*, ble*_-MBL_*, dfrA1, aadA1, bla*_OXA-10_*, catB8, ant(2'')-Ia, lnu(G*)
PM65	3	Chromosome	CP137233	4,035,582	/	*catA4, terZ, tet(J), catA1, bla*_TEM-1_ *, aph(3')-Ia, fosA3, bla*_CTX-M-65_ *, floR, sul2, aph (4)-Ia, aac (3)-IVa_1, aac(6')-Ib-cr_1, bla*_OXA-1_ *, catB3, arr-3, sul1, merC, merT, merR_Ps, aadA1, dfrA1*
		pPM65-2	CP137234	2,683	/	*qnrD1*
		pPM65-NDM_124k	CP137235	124,764	pPrY2001-like	*lnu(G), ant(2'')-Ia, catB8, bla*_OXA-10_*, aadA1, dfrA1, sul1, ble*_-MBL_*, bla*_NDM-1_*, aph(3')-VI, sul1, mph(E), msr(E), fosA3, merC, merT, merR_Ps, tet(B*)
PM1700	2	Chromosome	CP192503	3,995,082	/	*catA4, terZ, tet(J), aadA2, ere(A), dfrA32, aac (3)-IVa_1, aph (4)-Ia, sul2, floR, aadA1, dfrA1*
		pPM1700-NDM_54k	CP192504	54,761	IncN	*dfrA14, dfrA14, ble*_-MBL_ *, bla*_NDM-1_
PM8086	2	Chromosome	CP192505	3,999,338	/	*terZ, tet(J), aph(3'')-Ib, aph (6)-Id, floR, bla*_CTX-M-65_ *, fosA3, sul1, arr-3, catB3, bla*_OXA-1_ *, aac(6')-Ib-cr_1, aac (3)-IVa_1, aph (4)-Ia, sul2, bla*_NDM-1_ *, ble*_-MBL_ *, aadA2, dfrA12*
		pPM8086-NDM_156k	CP192506	156,223	pPrY2001-like	*floR, msr(E), mph(E), sul1, arr-3, bla*_NDM-1_ *, ble*_-MBL_ *, dfrA1, aadA1, bla*_OXA-10_ *, catB8, ant(2'')-Ia*
PM7752	2	Chromosome	CP191542	4,047,523	/	*catA4, terZ, tet(J), aadA2, ere(A), dfrA32, bla*_CTX-M-65_ *, fosA3, aph(3')-Ia, tet(C), sul2, aph(3'')-Ib, aph (6)-Id, floR, aadA1, dfrA1, bla*_TEM-1_ *, rmtB1, tet(G), floR2, sul1, qnrA9, aadA2, dfrA12*
		pPM7752-NDM_6k	CP191543	6,400	/	*arr-3, sul1, ble*_-MBL_ *, bla*_NDM-1_
PM8747	/	/	JBMERJ000000000	3,998,239	pPrY2001-like	*merC, merT, merR_Ps, tet(B), lnu(G), ant(2'')-Ia, catB8, bla*_OXA-10_ *, aadA1, dfrA1, sul1, ble_-MBL_, bla*_NDM-1_ *, aph(3')-VI, catA4, mph(E), msr(E), qnrD1, terZ, tet(J), aph(3')-Ia*
PM0259	/	/	JBMERK000000000	4,180,175	/	*tet(J), terZ, catA4, catA1, aadA1, dfrA1, sul1, ble*_-MBL_ *, bla*_NDM-1_ *, merR_Ps, merT, merC, bla*_CTX-M-14_ *, fosA3, dfrA17, aadA5, ant(2'')-Ia, merD, merB, merA, MerP_Gneg, merT, merR_Ps*
PM0460	/	/	JBMERL000000000	4,224,581	pPrY2001-like	*sul2, aadA1, dfrA1, catA4, tet(J), terZ, ble*_-MBL_*, bla*_NDM-1_*, aac (3)-IVa_1, aph (4)-Ia, merC, merT, merR_Ps, bla*_CTX-M-65_*, msr(E), mph(E), floR, arr-3, catB3, bla*_OXA-1_*, aac(6')-Ib-cr_1, aph(3'')-Ib, aph (6)-Id, catA1, bla*_TEM-1_*, lnu(F*)
PM3393	/	/	JBMERM000000000	4,225,321	IncN	*catA4, aadA1, dfrA1, bla*_TEM-1_ *, dfrA14, catA1, merC, merT, merR_Ps, qnrS1, ble*_O_ *, bla*_NDM-1_ *, tet(J), terZ*

^*a*^ The "/" in the Plasmid type indicates that it was not detected.

### Plasmid analysis and genetic structure

**Fig 2:**
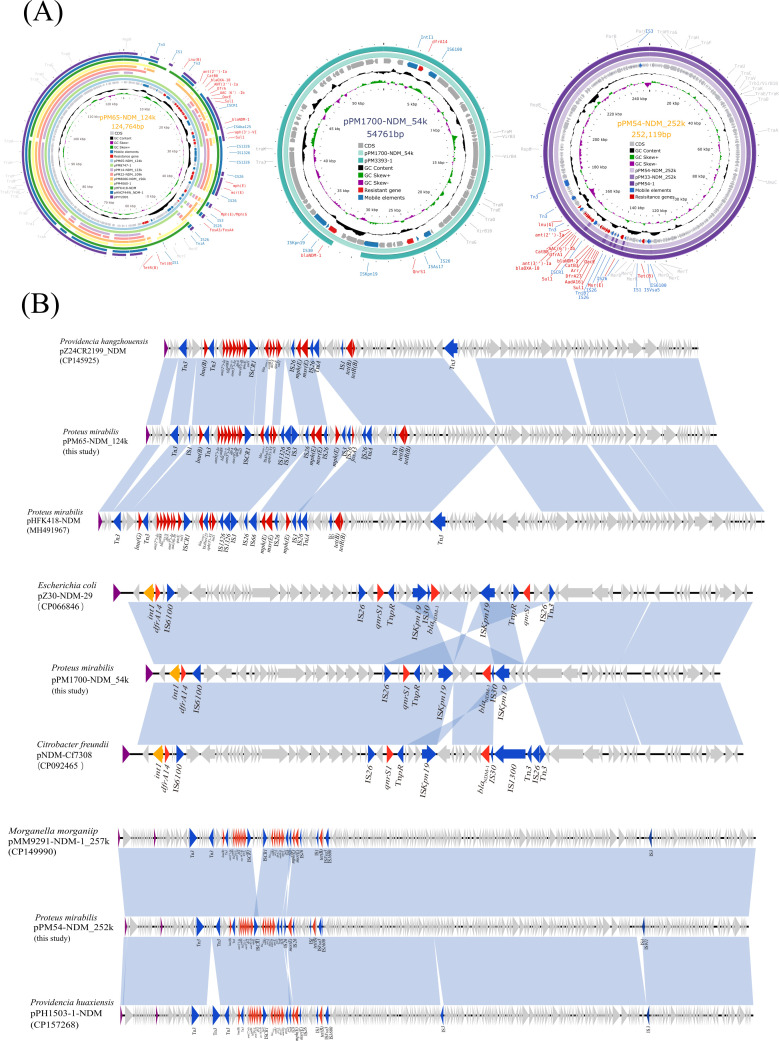
Genomic and molecular analysis of *bla*_NDM-1_-positive plasmids pPM65-NDM_124k, pPM1700-NDM_54k, and pPM54-NDM_252k. (**A**) Schematic diagram of pPrY2001-like plasmid, IncN plasmid, and pPM54-NDM_252k-like plasmid. (**B**) Comparative analysis of *bla*_NDM-1_-harboring plasmids: pPrY2001-like, IncN, and pPM54-NDM_252k-like plasmid, and shading in light blue indicates regions of homology (nucleotide identity ≥95%).

The complete sequences of *bla*_NDM-1_ carrying IncN plasmids (pPM1700-NDM_54k), pPrY2001-like plasmids (pPM14-NDM_123k, pPM22-NDM_109k, pPM8086-NDM_156k, pPM65-NDM_124k, and pPM8747-1) and pPM54-NDM_252k-like plasmid (pPM33-NDM_252k and pPM54-NDM_252k) were obtained (Fig. 2A). In the remaining four isolates (PM3393, PM8747, PM4060, and PM34), plasmid contigs were highly similar to the three *bla*_NDM-1_-bearing plasmids resolved with nanopore sequencing in this study. All tested plasmids, except the pPM54-NDM_252k-like type, conjugated successfully into *E. coli* J53. AST results showed that NDM-1-carrying *E. coli* transconjugants increased the MIC of carbapenems (meropenem, imipenem, ertapenem), cephalosporins (ceftazidime, cefepime), and beta-lactamase inhibitors (ceftazidime-avibactam) (Table 2).

The genome structure of pPrY2001-like plasmids consists of two main regions: the skeleton and helper module. Our pairwise comparison of pPrY2001-like plasmid scaffolds showed >97% nucleotide identity across >66% of their length, indicating conserved backbones (Fig. 2A and B). Subsequently, the pPM54-NDM_252k-like plasmid showed significant similarity to several plasmids found in clinical isolates in China, including *Morganella morganii* MM9291-NDM-1_257k (GenBank accession no. CP149990) and *Providencia huaxiensis* pPH1503-1-NDM (GenBank accession no. CP157268). Notably, pPM54-NDM_252k and MM9291-NDM-1_257k were isolated from the same tertiary hospitals in China (Fig. 2B) (16), indicating that *bla*_NDM-1_ may be transmitted between different species. IncN plasmid pPM1700-NDM_54k showed 99.99% identity and 100% coverage with pNDM-Cf7308 (GenBank accession no. CP092465) of *Citrobacter freundii* and pZ30-NDM-29 (GenBank accession no. CP066846) of *E. coli* isolated from China (100% coverage) was highly similar (Fig. 2B).

**Fig 3:**
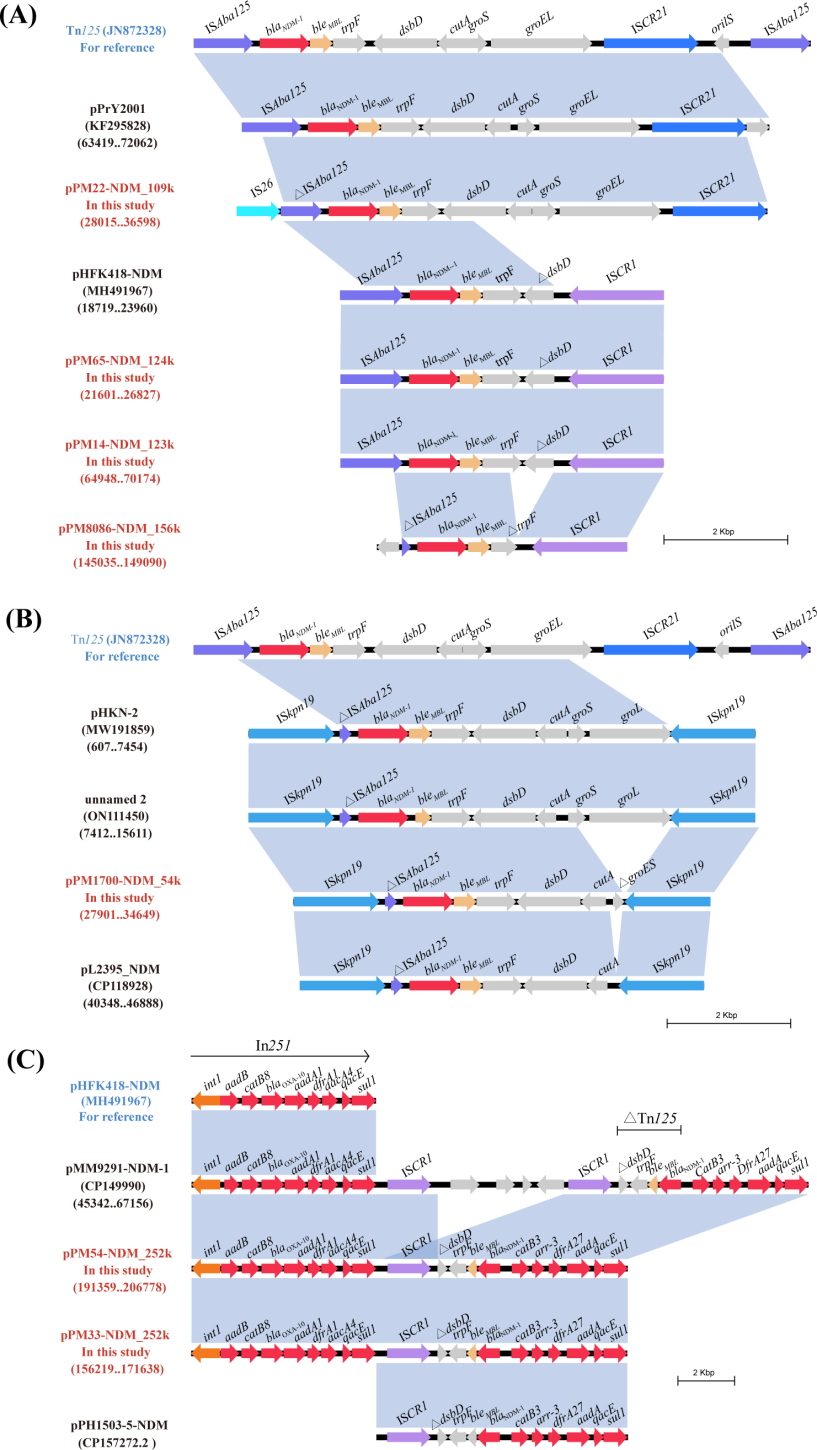
Core genetic background of *bla*_NDM-1_-harboring plasmids in *P. mirabilis*. The *bla*_NDM_ regions were compared with the related regions. Genes are indicated by the arrows. Genes, MGEs, and other features are colored according to their functional classification, and shading in light blue indicates regions of homology (nucleotide identity ≥95%). (**A**) Core genome analysis of pPrY2001-like plasmid. (**B**) Core genome analysis of IncN plasmid. (**C**) Core genome analysis of pPM54-NDM_252k-like plasmids.

The ΔTn*125* transposon carrying *bla*_NDM-1_ is usually located in the second variable region of the class I integrons. The structure of Tn*125* is IS*Aba125-bla*_NDM-1_-*ble*_MBL_-*trpF-dsbD-cutA-groES-groEL*-IS*CR21-oriIS*-IS*Aba125*, which was originally obtained from *Acinetobacter lwoffii* (17). The ΔTn*125* of the pPrY2001-like plasmids in this study can be roughly divided into three types. Compared to Tn*125*, the ΔTn*125* of pPrY2001 and pPM22-NDM_109k lacks the IS*Aba125* downstream of IS*CR27*. Additionally, ΔTn*125* from these two plasmids contained the following differences: IS*Aba125* upstream of ΔTn*125* in pPM22-NDM_109k was truncated, likely due to the insertion of IS*26* upstream of ΔIS*Aba125*. Another type of ΔTn*125*, with the structure IS*Aba125-bla*_NDM-1_-*ble*_MBL_-*trpF-*Δ*dsbD*, is found in pHFK418-NDM, pPM65-NDM_124k, and pPM14-NDM_123k. Notably, the type III ΔTn*125* located in the pPM8086-NDM_156k plasmid had a complete deletion of *dsbD*, and the upstream IS*Aba125* and *trpF* were truncated (Fig. 3A).

The derivative of Tn*125* was also present in pPM54-NDM_252k-like plasmid, with the structure *bla*_NDM_-*ble*_MBL_-*trpF-ΔdsbD*. The integron In251 is located upstream of ΔTn*125* in the pPM54-NDM_252k-like plasmid and contains multiple resistance genes, including *aadB*, *catB8*, *bla*_OXA–10_, *aadA1*, *dfrA1*, and *aacA4*. These findings suggest that Tn*125*-derived composite transposons are prevalent vectors that facilitate the spread of *bla*_NDM-1_ (Fig. 3C). These mobile structures likely facilitate clonal emergence and spread.

The genetic context of the *bla*_NDM-1_ gene in pPM1700-NDM_54k (IncN plasmid) is IS*Kpn19*-ΔTn*125* (ΔIS*Aba125-bla*_NDM-1_-*ble-trpF-dsbD-cutA-groES*)-IS*Kpn19,* where the *bla*_NDM-1_ gene is located downstream of a truncated IS*Aba125* element, which provides the −35 region of the promoter for *bla*_NDM-1_ (Fig. 3B) (18). This genetic environment has also been identified in the *E. coli* pHKN-2 plasmid, *Klebsiella pneumoniae* unnamed 2 plasmid, and *Citrobacter koseri* pL2395_NDM plasmid. Unlike pPM1700-NDM_54k, pHKN-2 and unnamed 2 possessed intact *groES* and *groL* within their ΔTn*125*, whereas *groES* was entirely absent in pL2395_NDM (Fig. 3B).

### SGI1 characterization

SGI1 is a site-specific integrated mobilizable element originally identified in *Salmonella enterica serovar Typhimurium* (19). SGI1 consists of a ~27.4 kb backbone with 28 ORFs from intSGI1 (S001) to resG (S027) plus S044 (19). Most SGI variants differ in *int1*; however, the SGI1-HLK group shows characteristic alterations in the backbone (12). Sequence analysis showed that four isolates of *P. mirabilis* (PM16, PM23, PM32, and PM8086) integrated SGI1 between the *thdF* and *hipB* chromosome genes. These SGI1 elements fell into three structural types.

**Fig 4:**
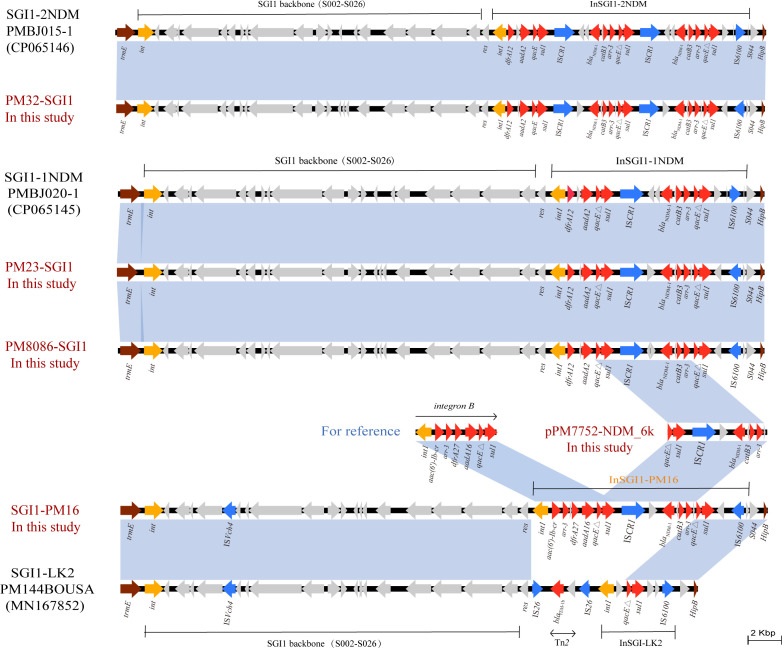
Comparative Schematic of *the bla*_NDM-1_-harboring SGI1 genomic islands. Genes are indicated by the arrows. Genes, MGEs, and other features are colored according to their functional classification, and light blue shading indicates (greater than or equal to) 95% nucleotide identity.

Comparative genomic analysis demonstrated that PM23-SGI1 and PM8086-SGI1 shared absolute nucleotide sequence homology (100% identity) with SGI1-1NDM, with PM32-SGI1 similarly displaying perfect sequence conservation with SGI1-2NDM. These two types of SGI1 were first reported in clinical *P. mirabilis* strains originating in China (10). The MDR region of SGI1-1NDM features a complex class 1 integron structure (InSGI1-1NDM), which is organized as 5′CS (*intI1*), VR1 (*dfrA12-orfF-aadA2*), 3′CS-1 (*qacEΔ1*/*sul1*), IS*CR1*, VR2 (*trpF-ble*_MBL_-*bla*_NDM-1_-*catB3-arr-3*), and 3′CS-2 (*qacEΔ1*/*sul1*). Compared to InSGI1-1NDM, InSGI1-2NDM carried two VR2 regions harboring *bla*_NDM-1_, along with adjacent IS*CR1* and 3′CS. Prior work showed that SGI1-2NDM variants with two *bla*_NDM-1_ copies exhibited higher carbapenem MICs and increased *bla*_NDM-1_ expression compared with single-copy variants and enhanced *bla*_NDM-1_ expression levels (2.1-fold increase) compared to single-copy variants (10) (Fig. 4).

Further comparison with existing SGI1s indicates that PM16 possesses a novel SGI1 structure, designated SGI1-PM16. SGI1-PM16 displayed SGI1-HKL-group backbone features, including an IS*1359* element with one end positioned within *traN*, consistent with the SGI1-HKL group. The MDR region of SGI1-PM16 harbors a class 1 integron (InSGI1-PM16) comprising 5′CS, VR1, 3′CS-1, IS*CR1*, VR2, and 3′CS-2. The VR2 structure is identical to that of InSGI1-1NDM described above, whereas the VR1 region matches the previously reported integron B (20), with the structure *aac(6’)-lb-cr5 -arr-3-dfrA27-aadA16* (Fig. 4). These findings suggest that SGI1 and its variants play a key role in the emergence and rapid spread of *bla*_NDM-1_ in *P. mirabilis*.

### IS*CR1* circular intermediates

IS*CR1* can generate IS*91*-like circular intermediates that undergo rolling-circle transposition via *oriIS* and *terIS* (21, 22). Whole-genome sequencing revealed that the genome of PM7752 contains a 6,400 bp circular fragment identical in structure to the 3′CS-1 (*qacEΔ1*/*sul1*), IS*CR1*, and VR2 (*trpF-ble*_MBL_-*bla*_NDM-1_-*catB3-arr-3*) regions of InSGI1-1NDM (Fig. 4). To further validate the circular nature of this sequence, junction-spanning primers were employed in PCR assays (YZ-F: AAGACATAATTGCTCACAG, YZ-R: ATGATCTAACCCTCGGTCTC), with amplicon size analysis confirming its circular conformation. These data suggest that *bla*_NDM-1_ can be mobilized via IS*CR1*-mediated circular intermediates that assemble into class 1 integron contexts, thereby facilitating further dissemination of *bla*_NDM-1_.

### Fitness effects

**Fig 5:**
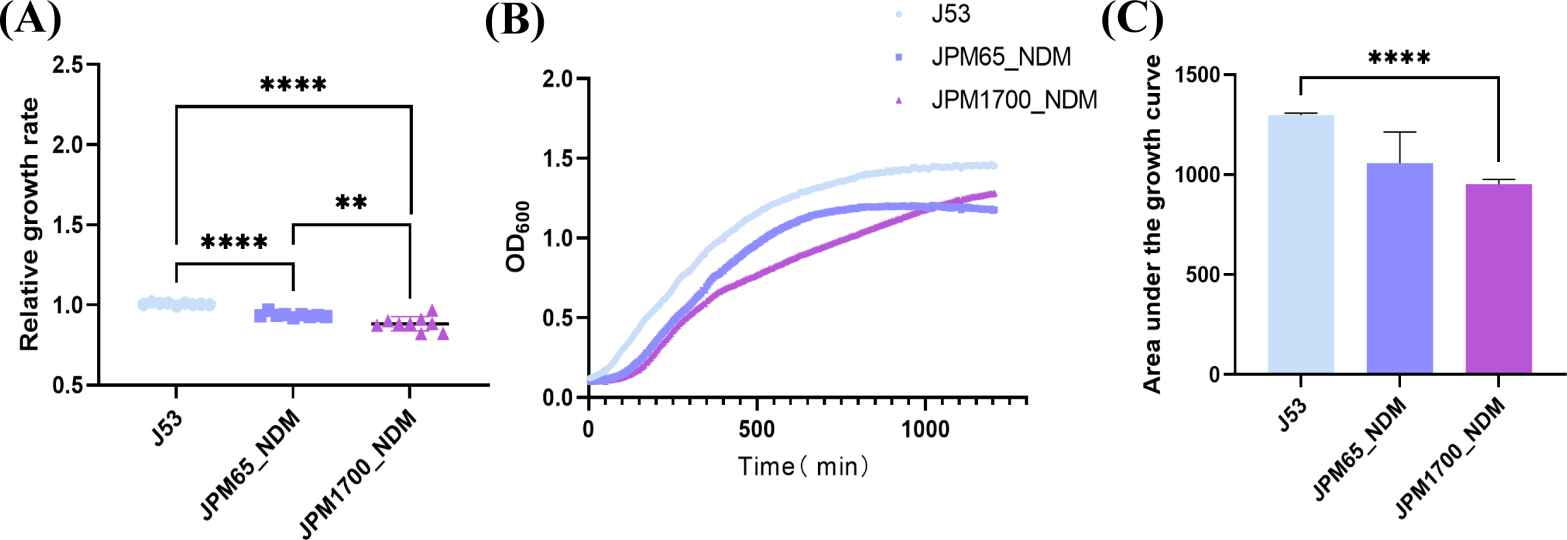
(**A**) Relative growth rates of strains JPM1700_NDM, JPM65_NDM, and J53. (**B**) Bacterial growth curves of strains JPM1700_NDM, JPM65_NDM, and J53. OD600, optical density at 600 nm. (**C**) Area under the growth curve for strains JPM1700_NDM, JPM65_NDM, and J53. Statistics: one-way ANOVA with post hoc tests across groups; two-sample *t*-tests for pairwise AUC comparisons; *P* thresholds as indicated **, *P <* 0.01; ****, *P <* 0.0001.

To assess the fitness cost associated with *bla*_NDM-1_-harboring plasmids, we performed comparative growth curve analyses of recipient *E. coli* strain J53 and transconjugants JPM1700_NDM and JPM65_NDM under antibiotic-free conditions. Quantitative analysis revealed that both NDM-1-producing transconjugants exhibited significantly impaired growth kinetics compared to the parental J53 strain (a 7.0%–12.2% reduction, *P* < 0.0001) (Fig. 5A), with lower final biomass (OD_600_) (Fig. 5B). Further evaluation of growth capacity using area under the curve (AUC) analysis showed JPM1700_NDM had ~26.8% lower AUC than J53 (956.2 ± 23.2 vs 1296 ± 10, *P* < 0.001), JPM1700_NDM showed a significant fitness reduction by AUC, whereas JPM65_NDM did not differ significantly from J53 by AUC despite modest rate/OD_600_ differences (Fig. 5C). These findings indicate that carriage of either the IncN-type plasmid or pPrY2001-like plasmid encoding *bla*_NDM-1_ imposes a measurable fitness burden on the host bacterium.

### Global phylogeography and phylogeny

**Fig 6:**
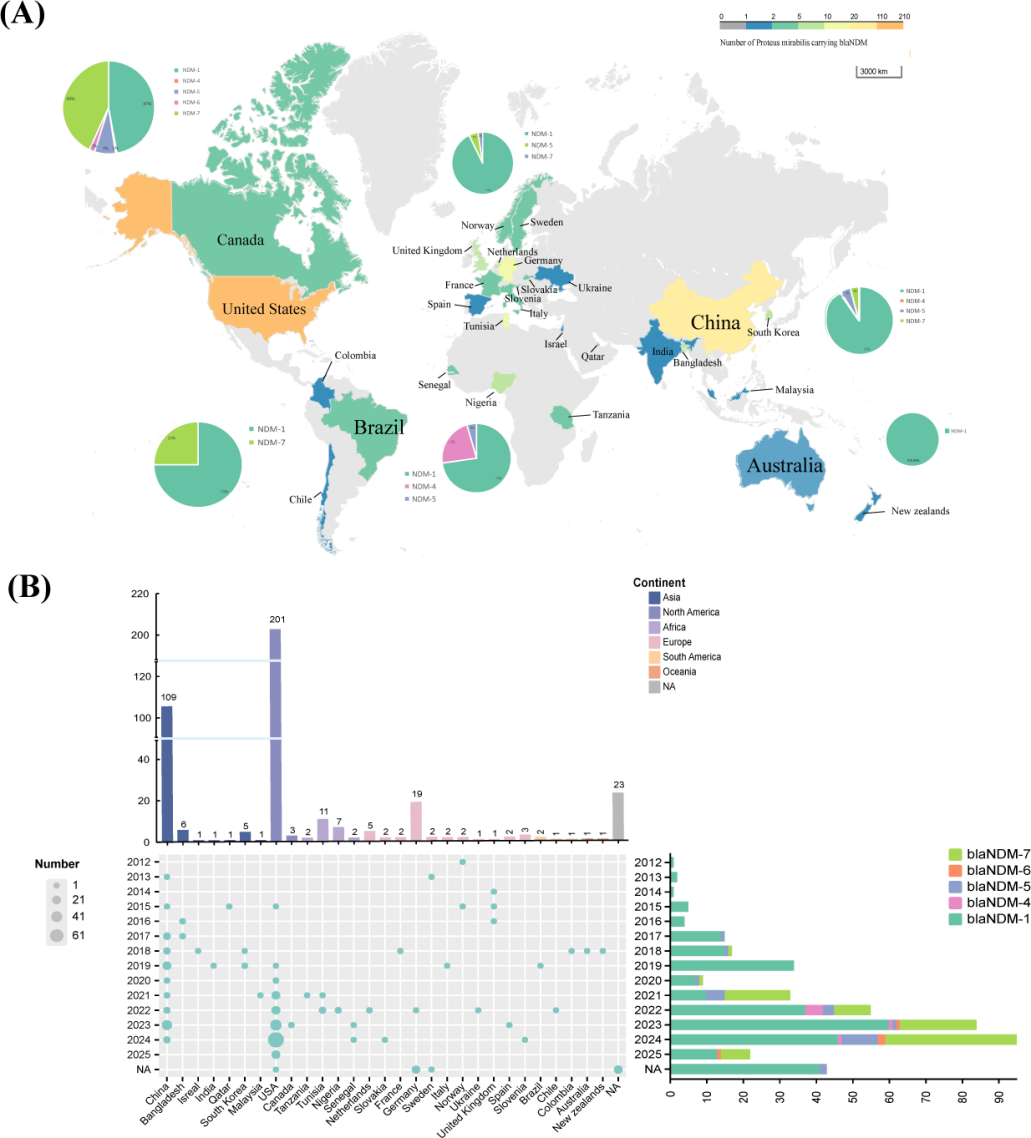
(**A**) Worldwide geographic distribution of *bla*_NDM_-producing CRPMs. Color gradient indicates isolate density. (**B**) Spatiotemporal distribution patterns of *bla*_NDM_-producing *P. mirabilis* clinical strains.

To investigate the phylogeographic relationships of NDM-producing CRPMs, basic information on 420 isolates was obtained from this study and the NCBI database (as of 21 April 2025). Most NDM-producing CRPMs were from North America (48.6%, 204/420), followed by Asia (29.5%, 124/420), Europe (9.8%, 41/420), Africa (5.2%, 22/420), and other regions (6.9%, 29/420); submissions increased over time (Fig. 6A and B). NDM-producing CRPMs have been reported in 29 countries worldwide, with the majority of isolates detected in the United States (47.86%, 201/420), followed by China (25.95%, 109/420), and Germany (4.52%, 19/420) (Fig. 6B).

**Fig 7:**
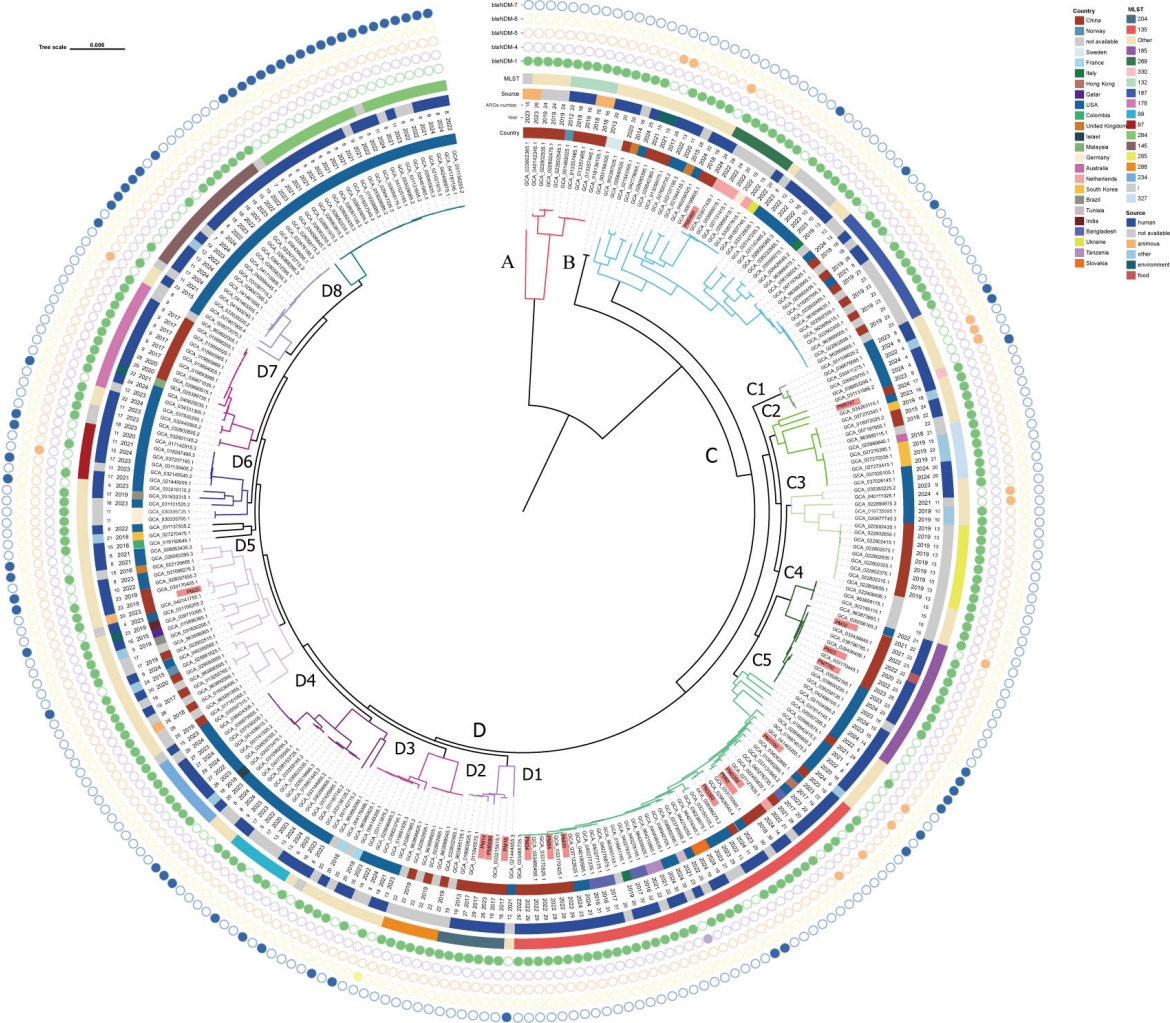
Core genome phylogeny of 420 *bla*_NDM_-producing CRPMs, comprising prospectively collected clinical isolates (denoted in red) and publicly available NCBI reference sequences.

Among *bla*_NDM_ variants, *bla*_NDM-1_ (69.1%, 290/420) and *bla*_NDM-7_ (22.6%, 95/420) predominated (>85% combined). Most of the NDM-1 producing CRPMs were from China (33.45%, 97/290), the United States (32.07%, 93/290), and European countries (13.1%, 38/290). Notably, the majority of NDM-7 producing CRPMs were distributed in the United States (92.63%, 88/95), and it has only a small distribution in China (5.26%, 5/95) and European countries (2.1%, 2/95) (Fig. 6A). MLST analysis showed that the 420 NDM-producing CRPMs could be divided into 93 distinct STs (Fig. 7). Among these, seven STs accounted for more than 3%, including ST135, ST284, ST145, ST185, ST269, ST178, and ST187. Among the CRPMs analyzed, ST135 emerged as the predominant sequence type, representing 14.05% (59/420) of the collection and exhibiting a broad geographic distribution across eleven countries. The second most prevalent lineage, ST284, accounted for 7.14% (30/420) of isolates and was restricted to China and the United States, followed closely by ST145 at 5.95% (25/420), which similarly demonstrated a bimodal distribution limited to these two nations (Table S1). Notably, CRPMs of all ST284 isolates carried *bla*_NDM-7_.

To trace the phylogenetic relationships between the *bla*_NDM-1_-carrying CRPMs in this study and other NDM-producing CRPMs globally, a phylogenetic tree was constructed using the assembled genomes of 420 NDM-producing CRPMs from this study and the NCBI database (Fig. 7). The phylogenetic tree showed that 420 strains carried multiple ARGs (ARGs number: 4–36), and 78.81% (331/420) of the strains carried ≥10 ARGs. The phylogenetic tree showed that the NDM-producing CRPMs could be divided into 4 clades, and the 16 clinical isolates in this study were distributed in clades B, C, and D.

## DISCUSSION

16 *bla*_NDM-1_-producing CRPMs from a tertiary hospital in China were resistant to third- and fourth-generation cephalosporins and carbapenems, which are commonly used clinically to treat infections caused by *Enterobacterales*. The prevalence of *bla*_NDM-1_-producing CRPMs presents a great challenge for clinical treatment not only because of its intrinsic resistance to polymyxins and tigecycline (23)but also because of its acquired resistance to ceftazidime-avibactam, which is one of the last options for the treatment of carbapenem-resistant Gram-negative bacteria (24). Despite the widespread prevalence of carbapenem resistance, fortunately, all 16 clinical CRPMs remained susceptible to cefiderocol and aztreonam-avibactam, which is consistent with previous studies (25, 26). Therefore, novel β-lactamase inhibitor combination therapies should be prioritized for clinical treatment.

The WGS revealed the widespread presence of ARGs in these CRPMs. Plasmid diversity is one of the main reasons for ARGs dissemination among bacteria (27). In our isolates, *bla*_NDM-1_ was located in a pPrY2001-like plasmid, an IncN plasmid, and a pPM54-NDM_252k-like plasmid; the latter being the first reported in *P. mirabilis* and was initially identified in *Morganella* spp. from the same hospital in China (16). Most of these plasmids were transferable, posing a risk for interspecies spread. The pronounced fitness costs observed in NDM-1-bearing transconjugants align with the metabolic burden theory of plasmid carriage (28). Notably, the IncN plasmid imposed more severe fitness defects than the pPrY2001-like variant, suggesting plasmid backbone-specific impacts on host physiology. Plasmid-specific fitness costs may shape persistence and spread dynamics.

In addition to plasmids, integrons and transposons also play pivotal roles in bacterial evolution and the dissemination of functional genetic elements, such as antimicrobial resistance genes. Through database screening and sequence analysis, we demonstrated that the transmission of *bla*_NDM-1_ in *P. mirabilis* is primarily mediated by the transposon ΔTn*125*. ΔTn*125*-mediated contexts were commonly associated with *bla*_NDM-1_ in our isolates, consistent with their role in dissemination (29), with most plasmids harboring truncated ΔTn*125*. Notably, these genetic elements all shared a conserved core structure: *bla*_NDM-1_-*ble*_MBL_-*trpF*. Previous studies have reported multiple copies of *bla*_NDM_ located on single plasmids or chromosomes (8, 16, 30), all of which were found in proximity to the IS*CR1* element. Similarly, in this study, the *bla*_NDM-1_ gene was also flanked by IS*CR1*. Experimental evidence confirms that IS*CR1* captures adjacent genes (often including antibiotic resistance determinants) through rolling-circle transposition initiated at its origin of replication (*oriIS*) (21). This element not only facilitates the interplasmid transfer of other resistance genes but also mediates the insertion of resistance genes into the chromosome. These findings highlight the significant role of IS*CR1* elements in the advanced stages of *bla*_NDM_ dissemination (29).

Chromosomal integration of resistance genes represents another important pathway that facilitates the dissemination of *bla*_NDM-1_. In this study, the *bla*_NDM-1_ gene in four *P. mirabilis* isolates was located on the chromosome within SGI1, specifically integrated between the *thdF* and *hipB* genes, which is consistent with previous reports (31). As previously reported, most SGI1 variants exhibit high conservation in their backbone regions. However, members of the SGI1-HKL group display characteristic modifications within the SGI backbone, where an insertion sequence, either IS*1359* or IS*Vch4*, replaces a 2.8 kb backbone segment extending from within *traN* (*S005*) to the interior of *S009* (12, 32). We identified a novel SGI1 variant, designated SGI1-PM16, which lacks the IS*26*-mediated Tn*2* region downstream of the res gene, compared to the previously reported variant SGI1-LK2. The VR2 structure in SGI1-PM16 was identical to those observed in InSGI1-1NDM and InSGI1-2NDM in this study. SGI1 is horizontally transmissible among bacteria, dependent on helper IncA/C-type plasmids for mobilization (33). The standard HKL deletion (*traN* to *S009*) does not impair transfer. SGI1-LK1, mobilized by an IncC plasmid, transferred to *E. coli* at 6.0 × 10⁻³ transconjugants per donor (12). This frequency aligns with prior reports (33). Given that SGI1-PM16 and SGI1-LK1 belong to the SGI1-HKL group with conserved architecture, these data collectively indicate transfer efficiency is governed by cis-acting regulatory elements outside the canonical HKL region.

Additionally, a 6,400 bp circular fragment with the same structure as VR2 was detected in isolate PM7752. Since IS*CR1* is a well-known mobile element that facilitates the dissemination of ARGs via rolling-circle transposition (22, 34), we hypothesized that the IS*CR1* element may mediate recombination of *bla*_NDM-1_-embedded VR2 at the *sul1* locus of class 1 integrons, thereby generating novel variants and promoting the spread of *bla*_NDM-1_ within SGI1. Notably, SGI1 of PM32 contains two VR2 regions with 100% nucleotide identity and carries two copies of *bla*_NDM-1_. Studies have demonstrated that *P. mirabilis* carrying dual copies of *bla*_NDM-1_ exhibit significantly higher MICs to meropenem, imipenem, and ertapenem compared to strains harboring a single copy. Additionally, *bla*_NDM-1_ expression levels were elevated by 2.63-fold in the dual-copy strains (10). This suggests that IS*CR1*-mediated rolling circle duplication may lead to the emergence of gene-enriched multiple copies (31), its high resistance to carbapenem antibiotics poses a new challenge for clinical treatment.

Our genomic epidemiological analysis revealed substantial clonal diversity among clinical CRPMs isolated from this hospital, comprising six distinct sequence types (ST135, ST185, ST204, ST218, ST269, and ST330). Notably, ST135 emerged as the predominant lineage (43.8%, 7/16). Furthermore, whole-genome SNP analysis identified three ST135 strains exhibiting high genetic relatedness, with a minimal pairwise SNP distance of <13, suggesting recent clonal expansion within this healthcare setting. Furthermore, ST135 strains formed a closely clustered group in the core genome phylogenetic tree, suggesting a potential intra-hospital clonal spread or common environmental exposure. The global phylogenetic tree further revealed the diversity of STs among the NDM-producing CRPMs in China, with ST135 being the dominant ST (11.92%). Phylogeographic reconstruction revealed distinct intercontinental dissemination patterns, with NDM-1-producing ST135 clones demonstrating epidemiological dominance in Asia (16/124, 12.9%), while NDM-7-carrying ST284 variants exhibited significant prevalence in North American isolates (28/204, 13.73%). Compared with *bla*_NDM-1_, NDM-7 hydrolytic activity can be higher than NDM-1 for some substrates, enhancing its resistance to carbapenem antibiotics (35). This difference might be one of the reasons for the regional distribution differences. These geographically stratified resistance patterns underscore the necessity for tailored infection control measures, including ST-specific surveillance and regionally optimized antimicrobial stewardship programs.

This study has several limitations. First, the cohort size (*n* = 16) limits power for some comparisons; second, single-center sampling may affect generalizability; and third, reliance on public genomes for global analysis may introduce sampling biases.

### Conclusions

This study demonstrates that *bla*_NDM-1_ disseminates through plasmids and chromosomal integration. We identified a novel SGI1-PM16 variant carrying a single *bla*_NDM-1_ copy. MGEs, such as Tn*125* and IS*CR1*, promote the horizontal transfer of resistance genes. IS*CR1*-mediated rolling-circle transposition likely facilitates transfer between plasmids and chromosome and can generate multi-copy resistance contexts. Both the IncN plasmid harboring *bla*_NDM-1_ and the pPrY2001-like plasmid imposed modest fitness costs in the absence of antibiotic selection. ST135 was the predominant sequence type among Chinese CRPM in our data set, while global NDM-producing CRPMs showed high genetic diversity (93 STs), reflecting adaptive evolution across different genetic backgrounds. This study provides essential data for understanding global CRPM epidemiology and informs region-specific containment strategies in China. Future infection control strategies should incorporate genomic surveillance, and combination therapies targeting NDM-1 warrant further exploration.

## MATERIALS AND METHODS

### Clinical bacterial isolates

Clinical isolates of *P. mirabilis* were obtained from a tertiary hospital in Zhejiang Province between January 2017 and September 2024. These isolates were initially characterized using Matrix-Assisted Laser Desorption/Ionization Time-of-Flight Mass Spectrometry (MALDI-TOF MS; bioMérieux, France). The MBL carbapenemase genes *bla*_NDM_, *bla*_KPC_, *bla*_IMP_, and *bla*_VIM_ were screened by PCR using specific primers from a previous study and confirmed by Sanger sequencing (36, 37).

### Conjugation assay and antimicrobial susceptibility testing

To investigate the transferability of *bla*_NDM-1_ in the CRPM isolates, a conjugation assay was performed. All CRPMs carrying carbapenemase genes were used as donors, and *E. coli* J53 was used as the recipient. The broth mating assay was performed as described previously, and mating mixtures were selected on Salmonella-Shigella agar containing 100 mg/L ampicillin and 200 mg/L sodium azide (38). Subsequently, transconjugants were validated by MALDI-TOF/MS, antimicrobial susceptibility testing, and PCR.

Antimicrobial susceptibility testing (AST) of 10 antimicrobial agents, including meropenem, imipenem, ertapenem, ciprofloxacin, cefepime, ceftazidime, cefiderocol, ceftazidime-avibactam, aztreonam-avibactam, and amikacin, was performed using a broth microdilution method with cation-adjusted Mueller-Hinton broth (CAMHB), while cefiderocol was used with iron-depleted CAMHB (16). Testing followed CLSI performance standards, and *E. coli* ATCC 25922 served as the QC strain (39).

### Whole-genome sequencing and analysis

WGS used Illumina HiSeq (short reads) and Oxford Nanopore MinION (long reads) at Zhejiang Tianke (Hangzhou, China), as previously described (40). Hybrid assemblies were generated with Unicycler v0.4.8 (41) and annotated using RAST (https://rast.nmpdr.org/) (42). ARGs and plasmid replicons were identified using ResFinder v4.6.0 (43) and PlasmidFinder v2.0.1 (44). BLAST from NCBI (https://blast.ncbi.nlm.nih.gov/Blast.cgi/) was used to identify similar sequences of plasmids and genes (45). Sequence comparisons were performed using BLASTn v2.4.0 (46) and visualized using Easyfig v2.2.3 (47) and Proksee (https://proksee.ca/) (48).

### Phylogenetic analysis

To trace the phylogenetic relationships between *bla*_NDM_-positive CRPMs from different sources, we used 16 clinically derived *P. mirabilis* genomes from this study and 404 assembled genomes carrying *bla*_NDM_ from different countries and sources in the NCBI Pathogen Database (https://www.ncbi.nlm.nih.gov/pathogens, accessed on 21 April 2025). Public CRPM genomes were included if assemblies were labeled *Proteus mirabilis*, carried any *bla*_NDM_ allele by ResFinder, and had country metadata; duplicates and metagenome bins were excluded. Core SNPs were called with Snippy (snippy-multi), trees inferred with FastTree (49) and further visualized using ChiPlot (https://www.chiplot.online/) (50). Heatmaps were utilized for the visualization of single-nucleotide polymorphisms (SNPs) using TBtools-II (51). Adobe Illustrator v27.9.1 was used to map the global geographic distributions.

### Growth rate determination

We determined the growth rates of transconjugants to investigate their fitness costs. As in a previous study (40), three separate cultures of J53, JPM65, and JPM1700 were cultured overnight and diluted in LB broth at 1:100, and then the equal sample was repeated three times into a flat-bottom 96-well plate. The plates were then incubated at 37°C. The optical density (OD) of each culture at 600 nm was measured using a Bioscreen C MBR machine (Oy Growth Curves Ab., Finland) at 5 min intervals for 20 h. The relative growth rate was estimated based on OD_600_ curves using an R script. Both the growth curve and the area under the curve (AUC) were derived from these OD_600_ measurements. The relative growth rates were then visualized using GraphPad Prism version 9. For three-group comparisons, we used one-way ANOVA with appropriate post hoc tests, and for pairwise AUC comparisons, we used two-sample *t*-tests (two-sided; *α* = 0.05).Statistical significance was set at *P* < 0.05.

## Data Availability

Complete chromosome and plasmid sequences for the indicated isolates are deposited in GenBank; accession numbers are provided in Table 3.

## SUPPLEMENTAL MATERIAL

Table S1 msystems.00758-25-s0001.xlsx Biosample information of global *P. mirabilis* genomes.

ASM does not own the copyrights to Supplemental Material that may be linked to, or accessed through, an article. The authors have granted ASM a non-exclusive, world-wide license to publish the Supplemental Material files. Please contact the corresponding author directly for reuse.
